# Unravelling the Transcriptome Profile of the Swine Respiratory Tract Mycoplasmas

**DOI:** 10.1371/journal.pone.0110327

**Published:** 2014-10-15

**Authors:** Franciele Maboni Siqueira, Alexandra Lehmkuhl Gerber, Rafael Lucas Muniz Guedes, Luiz Gonzaga Almeida, Irene Silveira Schrank, Ana Tereza Ribeiro Vasconcelos, Arnaldo Zaha

**Affiliations:** 1 Centro de Biotecnologia, Universidade Federal do Rio Grande do Sul (UFRGS), Porto Alegre, Brazil; 2 Programa de Pós-Graduação em Ciências Biológicas – Bioquímica, UFRGS, Porto Alegre, Brazil; 3 Laboratório de Bioinformática, Laboratório Nacional de Computação Científica (LNCC), Petrópolis, Rio de Janeiro, Brazil; 4 Departamento de Biologia Molecular e Biotecnologia, Instituto de Biociências, UFRGS, Porto Alegre, Brazil; Beijing Institute of Microbiology and Epidemiology, China

## Abstract

The swine respiratory ciliary epithelium is mainly colonized by *Mycoplasma hyopneumoniae*, *Mycoplasma flocculare* and *Mycoplasma hyorhinis*. While colonization by *M. flocculare* is virtually asymptomatic, *M. hyopneumoniae* and *M. hyorhinis* infections may cause respiratory disease. Information regarding transcript structure and gene abundance provides valuable insight into gene function and regulation, which has not yet been analyzed on a genome-wide scale in these *Mycoplasma* species. In this study, we report the construction of transcriptome maps for *M. hyopneumoniae*, *M. flocculare* and *M. hyorhinis*, which represent data for conducting comparative studies on the transcriptional repertory. For each species, three cDNA libraries were generated, yielding averages of 415,265, 695,313 and 93,578 reads for *M. hyopneumoniae, M. flocculare* and *M. hyorhinis*, respectively, with an average read length of 274 bp. The reads mapping showed that 92%, 98% and 96% of the predicted genes were transcribed in the *M. hyopneumoniae, M. flocculare* and *M. hyorhinis* genomes, respectively. Moreover, we showed that the majority of the genes are co-expressed, confirming the previously predicted transcription units. Finally, our data defined the RNA populations in detail, with the map transcript boundaries and transcription unit structures on a genome-wide scale.

## Introduction

*Mycoplasma hyopneumoniae*, *Mycoplasma flocculare* and *Mycoplasma hyorhinis* are the most important species that have been identified in the swine respiratory system [1], [2]. *M. hyopneumoniae* is the etiological agent of porcine enzootic pneumonia, whereas *M. hyorhinis*, in addition to being present in swine respiratory tracts, may cause swine serofibrinous to fibrinopurulent polyserositis and arthritis [3]. *M. flocculare* is also widespread in swine herds, but no disease has been associated with this species [4]. Based on a 16S rRNA sequence and genome comparison, it has been shown that *M. hyopneumoniae* and *M. flocculare* are phylogenetically closely related species [5], [6]. *M. hyopneumoniae* can adhere to the cilia of tracheal epithelial cells and cause damage. Although *M. flocculare* can also adhere to cilia, no important resulting damage has been observed, suggesting that *M. hyopneumoniae* and *M. flocculare* may possess different mechanisms of action involving the recognition of different receptor sites on the cilia [7]. In addition to the ability of *M. hyorhinis* to colonize other host sites [4], its potential role in human cancer development and acceleration to malignant phenotypes has been proposed [8]–[10].

Similar to other mycoplasmas, *M. hyopneumoniae*, *M. flocculare* and *M. hyorhinis* possess small genomes with limited biosynthetic potentials. Six *M. hyopneumoniae* genome strains have been sequenced [6], [11]–[14] in addition to four *M. hyorhinis* strains [10], [15]–[17] and one *M. flocculare* strain [6]. Although a significant amount of data has been produced by genome sequencing and comparative analyses in these species, very limited information related to transcriptional mechanisms and regulation is available for these organisms.

Analyses related to transcriptional unit (TU) organization, transcriptional regulation and promoter sequence composition have been performed on the *M. hyopneumoniae* 7448 genome [18]–[20]. Global assessments of the TU organization of the *M. hyopneumoniae* genome by both *in silico* and *in vitro* approaches have suggested that the ORFs are continuously transcribed (cotranscribed) in large clusters (TUs). The authors have predicted that each TU is transcribed in the same direction with no intervening gene transcribed in the opposite orientation [18]. Similar predicted TU organization to that of the *M. hyopneumoniae* genome have also been reported in the *M. flocculare* and *M. hyorhinis* genomes [6] in addition to that of *Mycoplasma pneumoniae* [21]. The advancement of knowledge of the whole transcriptome organization should contribute to the understanding of the unexplored mechanisms of mycoplasma transcriptional regulation.

Considering the importance of the three *Mycoplasma* species that inhabit the swine respiratory tract and the limited knowledge available regarding mycoplasma transcriptional regulation, we aimed to analyze the whole transcriptomes of *M. hyopneumoniae, M. flocculare* and *M. hyorhinis*. In this work, we have sequenced, analyzed and constructed single nucleotide resolution transcriptome maps for the three species. A comparative analysis of the whole gene expression profiles between these swine respiratory mycoplasmas was also performed. Furthermore, the organizations of the genomes into large TUs (polycistronic mRNA), as previously predicted, were estimated and compared between the three species.

## Materials and Methods

### Bacterial strains, culture conditions and RNA preparation

*M. hyopneumoniae* strain 7448, *M. flocculare* ATCC 27716 and *M. hyorhinis* ATCC 17981 were used in this study. The bacteria were grown in 25 ml Friis broth [22] at 37°C for 24 hours with gentle agitation in a roller drum.

Total RNA was isolated with the RNeasy Mini Kit (Qiagen, USA). For cell lysis, 0.7 ml of RNeasy Lysis Buffer (RLT buffer) in 0.134 M β-mercaptoethanol was used per cultivation flask. The purification was performed according to the manufacturer's instructions, with on-column DNase I digestion using the RNase-Free DNase Set (Qiagen, USA) and a second round of treatment with DNase I (Fermentas, USA). The absence of DNA in the RNA preparations was monitored by PCR. The extracted RNA was analyzed by gel electrophoresis and quantified using the Qubit system (Invitrogen, USA). RNA quality and integrity were determined by the evaluation of the RNA Integrity Number (RIN) using the Agilent 2100 Bioanalyzer (Agilent, USA). Values that were greater than or equal to 9.5 indicated sufficient quality.

The ribosomal RNA (rRNA) depletion was performed using Terminator 5′-phosphate-dependent exonuclease (TEX – Epicentre, USA), according to the manufacturer's instructions. Briefly, total RNA (10 µg) was combined with TEX 10X Reaction Buffer A, 20 U RiboGuard RNase Inhibitor, 10 U TEX (1 U/µg mRNA) and nuclease-free water in a final volume of 40 µl and incubated at 30°C for 60 minutes. The reaction was stopped with EDTA (0.5 M), and the mRNA was precipitated with 3 M sodium acetate and 2.5 volumes of ethanol. The mRNA was quantified using the Qubit system (Invitrogen, USA), and the effectiveness of the reaction was assessed by the Agilent 2100 Bioanalyzer (Agilent, USA). The absence of 16S and 23S rRNA in the posttreatment sample indicated a successful reaction.

### cDNA preparation and sequencing

The cDNA library preparation and pyrosequencing were performed using GS-FLX Titanium series reagents following the manufacturer's instructions (Roche Diagnostics, Germany). An equal amount of mRNA (200 ng) from each strain was used to synthesize the first and second strands of the cDNA, according to the *cDNA Rapid Library Preparation Method Manual GS FLX Titanium Series* (Roche Diagnostics, Germany). After the library construction, the samples were quantified using the Qubit system (Invitrogen, USA), and average fragment sizes were determined using the Agilent 2100 Bioanalyzer (Agilent, USA). Three cDNA libraries for each strain were generated, totaling nine libraries, which were sequenced using the Roche/454 GS-FLX system. Following the analyses of the reads, each of the triplicate sets of libraries was pooled.

### Assembly and mapping

For the transcriptome assembly, Newbler v2.8 was used with the default parameters. The FastQC software (http://www.bioinformatics.babraham.ac.uk/projects/fastqc/) was used for the read quality assessments, and the FASTX-Toolkit v0.0.13 (http://hannonlab.cshl.edu/fastx_toolkit/) was employed for trimming. Reads were aligned with Bowtie2 v2.1.0 [23] using the “sensitive” default option. The resulting alignment files were filtered with SAMtools v0.1.19 [24] to assess mapping quality (MQ; > = 10). BEDtools v2.17.0 [25] “multicov” was used to count reads mapped to specific genomic regions. Finally, matches were counted and the reads per kilobase per million (RPKM) computed [26]. Raw and normalized counts are available for each gene. To map the 454 reads, the *M. hyopneumoniae* 7448, *M. flocculare* ATCC 27716 and *M. hyorhinis* HUB-1 genomic sequences were used as references, and the rRNA sequences were filtered. The results were parsed, and GenBank (gb) format files were generated for analysis with the Artemis Release 10.5.2 software package [27], which included information about the coverage of each region and its respective gene identities. Circular genome plots were generated with DNAPlotter [28].

The Standard Flowgram Format (SFF) files of the sequence data generated in this study have been deposited in the Short Read Archive (SRA) database at NCBI under the accession number PRJNA255516.

### Analyses of TU structures

Expressed reads with coverage above background were mapped onto the annotated genes of *M. hyopneumoniae* 7448 (NC_007332), *M. flocculare* ATCC 27716 (NZ_AFCG00000000.1) and *M. hyorhinis* HUB-1 (NC_014448.1).

The operon assessment was based on published experimental data from *M. hyopneumoniae* 7448 [18]. Based on this study, the *M. flocculare* and *M. hyorhinis* TUs were predicted [6]. Thus, using the RNA-Seq results and in-house Perl scripts, we analyzed the predicted TUs for the three mycoplasma species, accounting for the following criteria: i) the previous prediction of the structure by Siqueira et al. [18] and Siqueira et al. [6] ii) the expression of all genes; iii) the transcription of the genes in the same direction; and iv) the expression of the intergenic regions between the genes. Overlapping pairs of such genes were joined together to identify large operon structures.

## Results and Discussion

### cDNA sequencing, assembly and mapping of *M. hyopneumoniae*, *M. flocculare* and *M. hyorhinis* transcriptomes

A total of 1,204,156 raw sequencing reads with an average length of 274 bp were generated using the Roche GS FLX Sequencing Platform. After cleaning and removing the adapters from each sequence in addition to low-quality sequences (quality scores <20) and rRNA sequences, a total of 683,385 high-quality reads were obtained and used for the *de novo* assembly. The cDNA libraries were constructed with total mRNA from *M. hyopneumoniae* 7448, *M. flocculare* ATCC 27716 and *M. hyorhinis* ATCC 17981. The RNA preparations from the three species were obtained from bacteria grown under the same culture conditions. Table 1 shows a detailed comparative analysis of each library assembly. To determine the transcribed regions in the genomes, we estimated the average coverage depths of the reads mapped per nucleotide/base. We used a pileup format, which produces a signal map file for the whole genome in which the alignment results (coverage depth) are represented reported per base. The resulting reads were mapped to the *M. hyopneumoniae* 7448, *M. flocculare* ATCC 27716 and *M. hyorhinis* ATCC 17981 genomes. The sequence coverage per base was subsequently plotted and visualized using the genome browsers Artemis and DNAplotter [27]–[28] (Figure S1).

**Table 1:** 454 sequencing and assembly data generated for *M. hyopneumoniae* (MHP), *M. flocculare* (MFL) and *M. hyorhinis* (MHR) transcriptome.

Library	MHP	MFL	MHR
Total reads	415265	695313	93578
Total bp	128786710	226650525	17499182
Total reads mapped bowtie2 -q10 (%)	390710 (94%)	647082 (93%)	80482 (86%)
Average length* (bp)	310.1	326	187
Total rRNA reads (16S/23S/5S)	232213	241658	46900
Contig number	1187	990	1120
Genes not mapping (% total genes)	63 (8%)	12 (2%)	31 (4%)
Total no. TU prediction	122	111	98
Total no. TU confirmed (% from total)	63 (52%)	67 (60%)	64 (65%)
Total no. TU partly confirmed (% from total)	34 (27%)	32 (30%)	22 (22%)
TU without any transcript gene	2	0	2
Total no. mC** prediction	40	45	34
Total no. mC transcript	36	43	32

*Average length of high-quality mRNA reads.

**Monocistronic gene.

The *M. hyopneumoniae* library allowed for the generation of 415,265 reads, of which approximately 94% mapped to the reference genome sequences of *M. hyopneumoniae* strain 7448. Most of the 695,313 reads (93%) from the *M. flocculare* library also mapped to the reference genome sequences of *M. flocculare* strain ATCC 27716. Similarly, 86% of the 93,578 reads from the *M. hyorhinis* library mapped to the reference genome sequences of *M. hyorhinis* strain HUB-1. The mapped *M. hyopneumoniae*, *M. flocculare* and *M. hyorhinis* genes represented 92%, 98% and 96% of the total predicted ORFs in these genomes, respectively (Table 1). The few unmapped genes were mainly identified as hypothetical genes with unknown functions (over 60%, considering the three transcriptome mappings). Full lists of the transcribed genes in *M. hyopneumoniae*, *M. flocculare* and *M. hyorhinis* under the culture conditions used in this study are presented as supplementary data (Table S1; Table S2; Table S3, respectively). Therefore, our results showed that the majority of genes were transcribed, demonstrating whole basal-level expression profiles for the mycoplasmas of the swine respiratory tract (Figure S1). The maps shown in Figure S1 represents the genomes (gray circles) and transcriptome maps (green circles) of *M. hyopneumoniae*, *M. flocculare* and *M. hyorhinis*, respectively, enabling the visualization of the high levels of gene coverage achieved by the cDNA assembly and mapping of the three *Mycoplasma* genomes.

As shown in Table 1, the total contig numbers were similar for the three libraries, and the coverages ranged from 1 to over 2,000 reads per contig, with most (62%) of the contigs covered by at least 15 reads. The contig length distributions were similar for the *M. hyopneumoniae*, *M. flocculare* and *M. hyorhinis* contigs (Figure S2). The majority of the contigs (approximately 25%) ranged in size from 200–500 bp, but many possessed lengths ranging from 1,000–3,000 bp (approximately 15%) (Figure S2).

Genome annotation refinement is an important aspect of a transcriptome analysis. The transcriptome map of *M. flocculare* allowed for the identification of five novel protein-coding genes that were not present in the initial annotation. The BLASTX search showed that they were homologous (similarities of 89% or more, sequence coverages of 100%) to genes from *M. hyopneumoniae* and other mycoplasma species. Three of these genes encoded hypothetical proteins, with the gene IDs MHP7448_0560, MHP7448_0709 and MHP7448_0138 in the *M. hyopneumoniae* 7448 genome, to which 127, 10 and 146 reads, respectively, were mapped in the *M. flocculare* transcriptome. Another novel identified gene encoded a lipoprotein with the gene ID MHP7448_0366 in *M. hyopneumoniae*, to which 31 reads were mapped in the *M. flocculare* transcriptome. A gene encoding a type III restriction-modification system: DNA methylase with the gene ID MHP7448_0386 in *M. hyopneumoniae* was also identified, to which 4 reads were mapped in the *M. flocculare* transcriptome.

According to the current annotation of the *M. flocculare* genome, the *ftsZ* gene is located in contig 04 and is positioned from nucleotides 8844–9491, which is located 647 bp downstream of a hypothetical gene (MF01093) in the opposite strand. However, the transcriptome map constructed in this study allowed for the identification of the incorrect annotation of the start codon of the *ftsZ* gene. The gene MF01093, with a highly expressed level (RPKM  = 139,138.8) (see Table S2), is in fact a part of the *ftsZ* gene. Thus, the *ftsZ* gene is positioned from nucleotides 8510–9505 (995 bp in length) in contig 04. This observation was confirmed by BLASTX comparisons against homologous genes in other *Mycoplasma* sp.

The transcriptome map of *M. hyorhinis* allowed for the identification of two novel genes. The first encoded a hypothetical protein, HUB-1, which was positioned from nucleotides 721079–721572 (with 12 associated reads). BLAST comparisons indicated that this gene shared 98% identity (with an e-value of 2e-85) with MYM_0631, which is a hypothetical protein that is positioned from nucleotides 719192–719647 in the *M. hyorhinis* GDL-1 genome and totals 453 bp in size. The second non-annotated gene was *rnpB*, to which 7,876 reads were mapped. The BLASTX analysis showed that it shared homologies (similarities of 98% or more, sequence coverages of 100%) with the genomes of other *M. hyorhinis* strains and other *Mycoplasma* species.

Additionally, in this work, we detected the endoribonuclease P (RNase P) ncRNA in the *M. hyorhinis* HUB-1 genome. RNase P, which is encoded by the *rnpB* gene, is a ribonucleoprotein complex that removes 5′ leader sequences from tRNA precursors during tRNA biosynthesis. RNase P is present in all cells and subcellular compartments that synthesize tRNA, but catalytic activity by the RNA alone has been demonstrated only for bacterial RNase P RNA. Bacterial RNase P RNAs have been separated into two main structural classes. Type A (*rnpA*) is the most common structural class, and type B (*rnpB*) is found in low G+C gram-positive bacteria [29]. *Mycoplasma* sp. genomes typically contain both classes of RNase P.

The availabilities of transcriptome maps for *M. hyopneumoniae*, *M. flocculare* and *M. hyorhinis* now allow for the detection of novel transcripts that have not been previously annotated in these genomes. Such transcripts include noncoding RNAs (ncRNAs), which were shown in other microorganisms to regulate important processes, such as pathogenesis, iron metabolism, and quorum sensing [30]–[32]. However, intergenic regions within TUs could represent portions of long expressed transcripts and be misclassified as ncRNAs. Therefore, these regions were excluded from this analysis. We manually analyzed the expressed intergenic regions located between the TUs to find evidence of novel ncRNAs. We identified 78, 130 and 72 putative novel ncRNAs in the *M. hyopneumoniae, M. flocculare* and *M. hyorhinis* transcriptomes, respectively (Table S4). Indeed, the BLASTX search provided evidence that these transcripts are ncRNAs. However, we believe that some of the putative ncRNAs that was detected in *M. flocculare* was possibly an artifact because this genome was not completely assembled.

**Figure 1:**
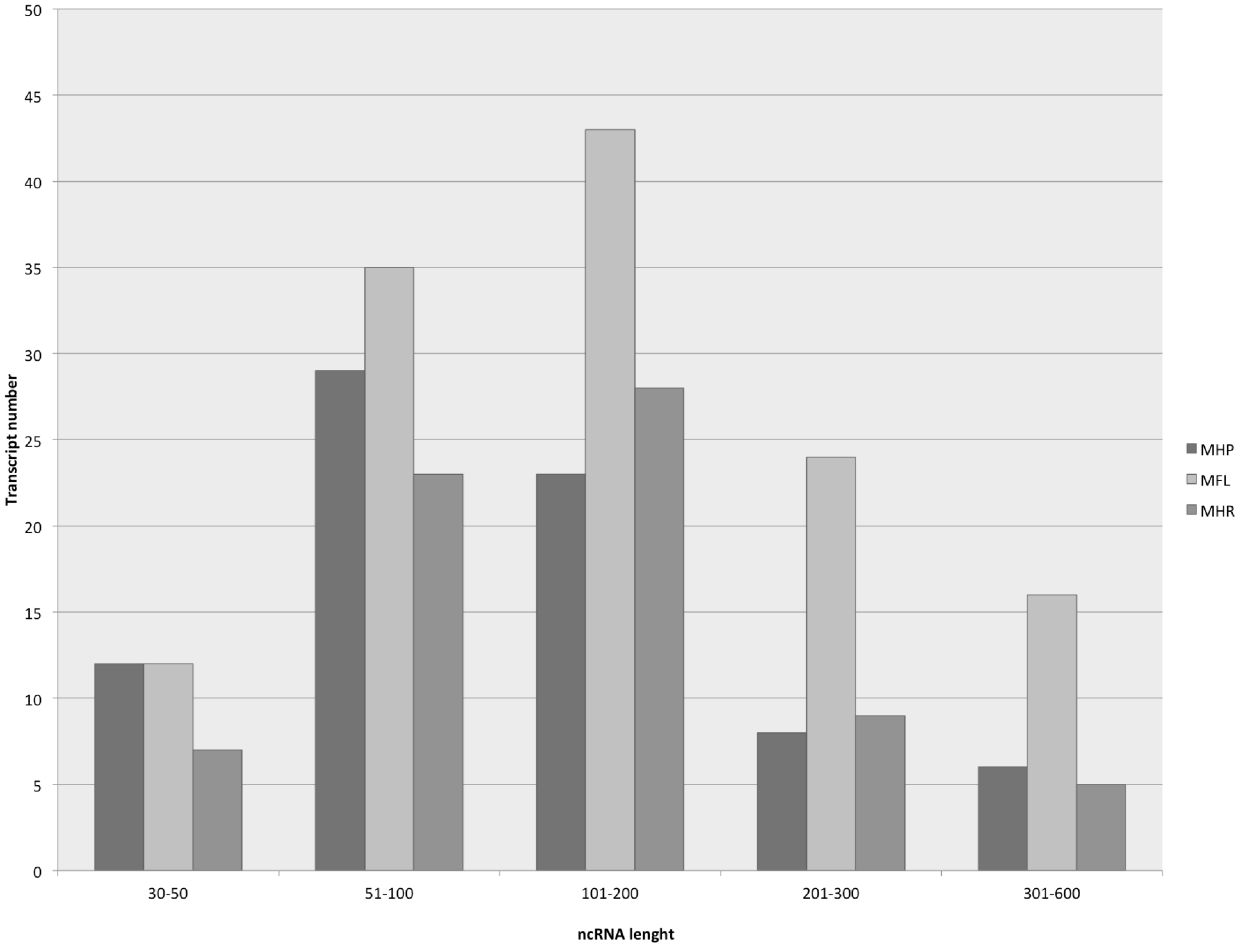
Noncoding RNA distribution lengths in *M. hyopneumoniae* (MHP), *M. flocculare* (MFL) and *M. hyorhinis* (MHR) transcriptome.

Although it is known that bacteria express sRNAs that are 50–500 nucleotides in length [33], we examined all reads with lengths ranging from 30–600 nucleotides (Table S4). The distributions of ncRNA lengths were very similar in the three organisms, and the majority of ncRNAs presented with lengths of between 51 and 200 nucleotides (Figure 1). This length variation may indicate the diversities of these elements, which may be related to RNA-based regulatory strategies. The putative ncRNAs detected here can represent some antisense RNA (asRNAs), but our approach was not designed to detect asRNAs. Further studies involving the determination of the strand specificity of expressed novel transcripts are necessary to fully characterize these regions.

In our *M. hyorhinis* transcriptome, we further identified a highly conserved ncRNA that is present in many bacteria, including *Mycoplasma* species, such as *M. hyopneumoniae* and *M. flocculare*, but not in any of the previously mapped *M. hyorhinis* genomes. This ncRNA is the signal recognition particle (SRP) ribonucleoprotein complex. The SRP RNA belongs to a group of small 4.5S RNAs that are found in bacteria, which direct the traffic of proteins within the cell and allow for their secretion. The SRP RNA, together with one or more SRP proteins, contribute to the binding and release of signal peptides [34].

### Comparative gene mapping of *M. hyopneumoniae*, *M. flocculare* and *M. hyorhinis* transcriptomes

The read distribution of the *M. hyopneumoniae* transcriptome showed that 92% of the predicted genes possessed significant alignments with one or more sequences (Table S1). A detailed analysis demonstrated that approximately 80% of the mapped genes have RPKM ≥5. Some of genes with the best RPKM values are summarized in Table 2. Notably, the products of the genes with the highest expression level were mainly associated with basal metabolism and also included chaperone proteins, adhesins, surface proteins and transporters; the highest RPKM values was 159,921.4 (corresponding to 30,423 reads) that match to the transcript of the MHP_0104 gene (*rnpB*), which encodes the RNA component of RNase P. Other genes that were also associated with high RPKM values included those encoding the cell division protein MraZ, S-adenosyl-methyltransferase, cell division protein FtsZ, p216 surface protein and hexosephosphate transport protein.

**Table 2:** Genes mapped with the highest number of transcript reads in the *M. hyopneumoniae* genome.

Locus tag	Gene Name	Product	Reads Count	RPKM
MHP0104	rnpB	RNAse P RNA	30423	159921.4
MHP0391	mraZ	Cell division protein mraZ	28537	156810.5
MHP0392	mraW	S-adenosyl-methyltransferase	33219	90347.1
MHP0393	ftsZ	Cell division protein ftsZ	12076	29739.5
MHP0496	-	p216 surface protein	25208	10796.4
MHP0136	-	Hexosephosphate transport protein	3788	6308.9
MHP0115	pdhA	Pyruvate dehydrogenase E1-alpha subunit	1745	3758.8
MHP0137	ldh	L-lactate dehydrogenase	1416	3619.6
MHP0067	dnaK	Chaperone heat shock protein 70	2113	2839.9
MHP0377	-	Conserved hypothetical protein	1164	2678.7
MHP0116	pdhB	Pyruvate dehydrogenase	885	2133.9
MHP0225	-	Methylmalonate-semialdehyde dehydrogenase	1232	2030.9
MHP0488	-	Conserved hypothetical protein	474	1886.1
MHP0226	iolC	Myo-inositol catabolism protein	733	1746.6
MHP0411	-	Hypothetical protein	231	1608.6
MHP0376	sgaT	Transport protein sgaT	1061	1452.6
MHP0096	tpx	Thiol peroxidase	279	1374.2
MHP0721	-	Hypothetical protein	141	1372.2
MHP0713	-	Conserved hypothetical protein	218	1344.2
MHP0227	iolB	Myo-inositol catabolism protein	433	1300.2
MHP0427	efp	Elongation factor EF-P	298	1294.2

The transcript mapping of the *M. flocculare* genome revealed that 98% of the predicted genes significantly aligned to one or more sequences of the *M. flocculare* transcriptome (Table S2). A detailed analysis demonstrated that approximately 94% of the mapped genes have RPKM ≥5. Some of genes with the best RPKM values are summarized in Table 3, and similar to the findings observed for *M. hyopneumoniae*, those genes with high numbers of reads were related to basal metabolism, chaperone proteins, adhesins and unknown products. Similar to *M. hyopneumoniae*, in *M. flocculare*, the *mraZ* and *ftsZ* gene, which encode cell division proteins, present a higher transcriptional level compared with the other genes. A gene that is exclusive of *M. flocculare*, encoding hypothetical protein (MF01463), and another four hypothetical genes (MF00750, MF00857, MF01460 and MF01461) with orthologs in the *M. hyopneumoniae* genome also presented with high RPKM values (Table 3).

**Table 3:** Genes mapped with highest number of transcript reads in the in *M. flocculare* genome.

Contig	Locus tag	Gene name	Product	Reads Count	RPKM
AFCG01000004	MF01423	mraZ	Cell division protein MraZ	138839	449493.1
AFCG01000004	MF01101	mraW	S-adenosyl-methyltransferase	148467	238708.1
AFCG01000004	MF01095	ftsZ	Cell division protein ftsZ	74695	218041.4
AFCG01000003	MF01463	-	Hypothetical protein	2330	12600.9
AFCG01000014	MF00857	-	Hypothetical protein	2564	6011.1
AFCG01000014	MF01460	-	Hypothetical protein	758	4624.9
AFCG01000014	MF01461	-	Hypothetical protein	824	4309.4
AFCG01000001	MF00750	-	Hypothetical protein	2863	3881.9
AFCG01000004	MF00691	tuf	Elongation factor EF-P	1154	2905.8
AFCG01000008	MF01167	dnaK	Chaperone dnaK heat shock protein 70	3064	2426.3
AFCG01000005	MF00591	-	Adenine phosphoribosyltransferase	846	2340.8
AFCG01000014	MF00861	mgtE	MG2 + transport protein	1718	1675.5
AFCG01000005	MF01362	tpx	Thiol peroxidase	503	1459.7
AFCG01000005	MF00043	-	Dihydrolipoamide acetyltransferase	876	1358.0
AFCG01000007	MF01334	-	Translation initiation factor IF-3	318	1339.3
AFCG01000006	MF00378	ptsH	Phosphocarrier protein HPr	247	1306.1
AFCG01000001	MF01384	-	PTS system enzyme IIB component	266	1305.1
AFCG01000007	MF00172	tig	Trigger factor	1225	1292.7
AFCG01000005	MF00585	pdhA-1	Pyruvate dehydrogenase E1-alpha subunit	1016	1289.4
AFCG01000007	MF00118	infC	Translation initiation factor IF-3	266	1229.1

Surface proteins are potentially related to bacterial pathogenesis. The *M. hyopneumoniae* colonization of respiratory ciliated epithelium relies specifically on the expression of two functionally redundant adhesin families that are paralogs of P97 and P102. The *M. hyopneumoniae* gene encoding P216 adhesin (MHP7448_0496) presented with a significant amounts of transcripts (RPKM  = 10,796.4) (Table 2), similarly to previous studies that predicted this protein to be a highly expressed cilium adhesin in *M. hyopneumoniae* [35], [36]. P216 is a proteolytically processed cilium and heparin binding protein [35], and recent data have highlighted the importance of endoproteolysis as a means of generating surface diversity in *M. hyopneumoniae* [36]. Other genes encoding surface proteins, such as MHP7448_0656 (lipoprotein p65), MHP7448_0198 (protein p97-copy 1) and MHP7448_0497 (p76 membrane protein), which are possibly involved in host cell adhesion, were also among the genes with high transcriptional level (Table 2).

Previous analyses have shown that the orthologs of *M. hyopneumoniae* genes encoding MHP7448_0198 (protein p97-copy 1) and MHP7448_0199 (protein p102-copy 1) are the only adhesin genes absent in the *M. flocculare* genome [6]. In the current study, different RPKM amounts, that suggest different expression level, was found in other genes encoding adhesin-like proteins in the *M. flocculare* genome compared with those of the *M. hyopneumoniae* transcriptome (Table 4), and even higher numbers of reads mapped to MF00472 (protein p97-copy 2, with RPKM  = 1,089.7) (the *M. hyopneumoniae* ortholog was MHP7448_0108 with RPKM  = 234.7) and MF00475 (protein p102-copy 2, with RPKM  = 151.8) (the *M. hyopneumoniae* ortholog was MHP7448_0107 with RPKM  = 51.1). The finding of higher numbers of reads mapping in the *M. flocculare* adhesins compared with those of the *M. hyopneumoniae* orthologs was unexpected (see Table 4), but according to a previous gene organization and location analysis, the regions containing these orthologs in *M. flocculare* display inversions and rearrangements in contrast with those of *M. hyopneumoniae* [6], which may be related to changes in the products' functionalities [46]. Furthermore, the p97-copy 2 protein of *M. flocculare* lacks the R1 domain [6]. Specific cleavages in repeat regions, which are designated as R1 and R2, play key roles in *M. hyopneumoniae* adherence [39], [44]. These R1 and R2 repeats are absent from the *M. flocculare* p97-like protein and its orthologs from *M. hyopneumoniae* (p97-like adhesin). Thus, even with the high read numbers of some of the adhesins that were observed in the current study, *M. flocculare* possesses a weak adhesion ability [7], which may be due to the variants of these proteins that lack the functional domains that are associated with adhesion capacity or antigenicity [38], [39], [44].

**Table 4:** Summary of adhesins associated to pathogenicity, genome organization and transcription profile comparison.

Product	MHP* Transcriptome RPKM (N° reads)	MFL* Transcriptome RPKM (N° reads)	MHP cotranscription	MFL cotranscription	ID MHP_7448	ID MFL	MHP x MFL genome location comparison	Target binding (reference for studies in MHP strain 232)
P97-like	3.9 (5)	14.9 (32)	no	yes	MHP7448_0272	MF00620	inverted	heparin, fibronectin, plasminogen [37]
P116 or P102-like	4.9 (6)	25.7 (55)			MHP7448_0271	MF00623	inverted	porcine cilia, fibronectin, plasminogen [38]
P97 - copy 2	234.7 (306)	1,089.7 (2,434)	yes	yes	MHP7448_0108	MF00472	inverted	porcine cilia, heparin, fibronectin [39]
P102 - copy 2	51.1 (60)	151.8 (341)			MHP7448_0107	MF00475	inverted	NA
P216	10,796.4 (25,208)	72.5 (278)	yes	yes	MHP7448_0496	MF00848	inverted	porcine cilia, heparin [35]
P159, P110 or P76	333.1 (586)	265.2 (559)			MHP7448_0497	MF00844	inverted	heparin [40]
P97 - copy 1	458.7 (619)	NP	yes		MHP7448_0198	NP	NP	porcine cilia, heparin [41]
P102- copy 1	26.8 (30)	NP			MHP7448_0199	NP	NP	heparin, fibronectin, plasminogen [42]
Hypothetical protein	79.2 (119)	32.8 (81)	yes	yes	MHP7448_0662	MF01055	inverted	porcine cilia, glycosaminoglycan [43]
P146	105.3 (173)	175.5 (448)			MHP7448_0663	MF01050	inverted	porcine cilia, heparin, and plasminogen [44]
lipoprotein	269.8(328)	165.4 (326)	yes	yes	MHP7448_0373	MF00741	conserved	porcine cilia, heparin [45]
Hypothetical protein	56.5 (67)	21.4 (42)			MHP7448_0372	MF00739	conserved	porcine cilia, heparin [45]
P95	11.3 (16)	21.5 (49)	yes	yes	MHP7448_0099	MF00492	inverted	NA
P60	54.6 (36)	76.5 (86)			MHP7448_0353	MF01236	inverted	NA

NA - not analyzed; NP - not present.

*MHP - *M. hyopneumoniae* 7448; MFL - *M. flocculare* ATCC 27716.

Interestingly, the expression level of MHP7448_0198 (p97-copy 1), which has no ortholog in *M. flocculare*, was elevated, as shown in Tables 2 and 4. Some studies have shown that p97 and p102-copy 1 are important to the pathogenic capacity of *M. hyopneumoniae* [41], [42]. Moreover, a significant number of reads (25,208 reads; RPKM  = 1,0796.4) mapped to cilium adhesin P216, which was highly expressed in *M. hyopneumoniae*, compared to the *M. flocculare* ortholog with 278 reads (RPKM  = 72.5) (Table 4). The P216 protein is cleaved, generating the heparin-binding proteins p120 and p85, which bind to porcine cilia and are recognized by serum antibodies. These two proteins play significant roles in the interactions of *M. hyopneumoniae* with host cells by playing immunoreactive roles in the humoral immune response of swine during the natural course of infection, and they are important components of the surface architecture of *Mycoplasma* cells [35], [36]. In summary, the p97-copy 1 and P216 adhesins may be related to the pathogenic capacity of *M. hyopneumoniae* based on their presences and expression patterns.

The transcriptome analysis of *M. hyorhinis* was performed with the strain *M. hyorhinis* ATCC 17981. Because the genome of this strain was not available, the transcriptome assembly and mapping was based on the *M. hyorhinis* HUB-1 genome. The percentage of reads mapped (86%) was the lowest among the three mycoplasma cDNA libraries (Table 1). This result indicates that an assembly with a different genomic strain can produce inaccurate mapping, especially in intergenic regions. Ninety-six percent of the predicted *M. hyorhinis* HUB-1 genes were identified in our transcriptome (Table 1), and approximately 95% of the mapped genes have RPKM ≥5. Some of genes with the best RPKM values are summarized in Table 5. The gene encoding the MraZ protein was the gene with more represented expression level in *M. hyorhinis* transcriptome (RPKM  = 8,637.4) (Table 5), followed by other genes that were mainly related to basal metabolism, including hexosephosphate transport protein, *gap, pdhA*, *rdhB*, and *tuf*. Somewhat similar to the other two transcriptomes, the *mraW* gene, which encodes S-adenosyl-methyltransferase, was associated with a significant RPKM measurement (RPKM  = 1,456.1) (Table 5). The genes *vlpF*, *vlpE*, *vlpB* and *vlpG* which encode variant surface proteins that are exclusive to *M. hyorhinis*, in addition to MHR_0162 (surface antigen) also were aligned by expressive numbers of reads.

**Table 5:** Genes mapped with the highest number of transcript reads in *M. hyorhinis* genome.

Locus tag	Gene Name	Product	Reads Count	RPKM
MHR_0351	mraZ	Protein mraZ	276	8637.4
MHR_0432	-	Hexosephosphate transport protein	586	5619.3
MHR_0339	vlpF	Variant surface antigen F	64	3900.3
MHR_0338	vlpE	Variant surface antigen E	150	3405.6
MHR_0611	gap	Glyceraldehyde 3-phosphate dehydrogenase C	226	3134.0
MHR_0348	vlpB	Variant surface antigen B	168	3100.1
MHR_0162	-	Surface antigen	296	3060.2
MHR_0343	vlpG	Variant surface antigen G	159	2910.8
MHR_0517	pdhA	Pyruvate dehydrogenase E1-alpha subunit	224	2826.9
MHR_0600	-	Probable exported protein	53	2530.7
MHR_0038	-	Hypothetical protein	21	2431.6
MHR_0560	tuf	Elongation factor Tu	184	2114.7
MHR_0516	pdhB	Pyruvate dehydrogenase E1 component beta subunit	150	2111.7
MHR_0629	-	Membrane protease subunitC- like protein	50	1767.8
MHR_0460	-	Hypothetical protein	131	1728.6
MHR_0675	-	Hypothetical protein	36	1667.4
MHR_0035	nox	NADH oxidase	159	1604.4
MHR_0006	-	Putative MgpA-like protein	108	1534.4
MHR_0352	mraW	Ribosomal RNA small subunit methyltransferase H	94	1456.1

*M. hyorhinis* contains an exclusive variable lipoprotein (Vlp) system that constitutes its major coat proteins [47] and provides a strategy for evading the host immune system. Different *M. hyorhinis* strains carry variable numbers of *vlp* genes [47]. *M. hyorhinis* HUB-1 is characterized by the presence of seven *vlp* genes displayed in the order 5-*vlpD*-*vlpE*-*vlpF*-insertion sequence (IS)-*vlpG*-*vlpA*-IS-*vlpB*-*vlpC*-3′ [15]. Although all seven *vlp* genes were identified in the *M. hyorhinis* transcriptome map, the transcript numbers mapping to the different *vlp* genes were variable. *vlpF*, *vlpE* and *vlpB* were mapped by more reads compared with *vlpG*, *vlpD* and *vlpC* (see Table S3). No reads were mapped to *vlpA* in this analysis. Previous reports have shown that structural variations affect the abundances and functionalities of Vlps on the mycoplasma surface [48], but the full consequences of these variations are yet to be understood. An organism carrying multiple *vlp* genes has the capacity to generate a large number of variants expressing antigenically distinct Vlp products, either alone or in combinatorial mosaics on the cell surface. Citti et al. [49] argued strongly for the associations of specific functions with each product, which are presumably selected for in the natural host niche. Most likely, the ability of *M. hyorhinis* to evade the host immune system is related to the variable expressions of the *vlp* genes, and therefore, future research may improve knowledge regarding the variation of Vlps in *M. hyorhinis*.

Cell wall biosynthesis by gram-positive and gram-negative organisms is a complex process that involves numerous proteins and has both cytoplasmic and membrane components [50]. The processes of cell wall biosynthesis and cell division are therefore tightly coupled. The protein products of the *mraW, ftsH, ftsY, ftsZ* and *mraZ* genes are involved in bacterial cell wall biosynthesis and/or cell division [51] and are the currently representative proteins in swine respiratory mycoplasmas. All of these genes were transcribed in the three mycoplasma maps with abundant transcript counts (Table 2; Table 3; Table 5).

Abundant numbers of transcripts were identified in the three transcriptome maps that aligned to S-adenosyl-methyltransferase-associated genes, that participate in cell wall biosynthesis (*mraW*) and cell division (*mraZ*). Notably, the gene *ftsZ*, which encodes a cell division-related protein, is among the genes mapped with the largest RPKM amounts in the *M. hyopneumoniae* and *M. flocculare* transcriptomes (Table 2; Table 3). However, the transcriptional levels of this gene were lower in *M. hyorhinis* (RPKM  = 839.9). Most bacteria produce the tubulin homolog FtsZ [52], which forms a cytoskeletal filament that is essential for membrane constriction and the coordination of septal peptidoglycan synthesis [53]. Although mycoplasmas possess a gene encoding FtsZ and some evidences suggested this gene as essential to these organisms [54]. However, more recently, it was experimentally demonstrate that *ftsZ* is non-essential for cell growth and in the absence of the FtsZ protein, *M. genitalium* can manage feasible cell divisions and cytokinesis using the force generated by its motile machinery [55]. Furthermore, in *M. pneumoniae*, very low levels of both *ftsZ* mRNA and FtsZ protein are present [56], supporting the hypothesis that mycoplasmas do not actually use FtsZ solely for cell division and revealing a potentially unusual function of this protein.

Another functionally related group of transcripts with significant RPKM amounts encoded heat-shock proteins (HSPs) (Table S1; Table S2; Table S3). HSPs are induced by environmental stress and play important roles in stimulating both the host innate and adaptive immune responses [57], [58]. These proteins can be classified into six families as follows: the large-molecular-weight HSP family, HSP90 family, HSP70 family, HSP60 family, small-molecular-weight HSP family, and ubiquitin [59]. HSP70 has been extensively studied and is known to function as a molecular chaperone, anti-cell apoptosis agent, and antioxidant and to induce immune responses, improve stress tolerance, and promote cell proliferation, cytoskeleton formation, and repair [60]. Both HSP70 and HSP60 induce immune responses that protect hosts against bacterial and mycoplasmal infections [61]–[63]. Furthermore, it has been shown that monoclonal antibodies generated against a portion of *M. hyopneumoniae* HSP70 are capable of blocking the growth of *M. hyopneumoniae* [64]. In the three transcriptomes we observed high numbers of reads that were mapped to the DnaK gene (HSP70), which forms a chaperone protein complex with the DnaJ and GrpE genes [63] Additionally, DnaJ and GrpE mapping also resulted in a significant number of reads. HSP60 (GroEL) was absent in the three mycoplasmas of the swine respiratory tract [6], [12], [15].

A comparative genome analysis between *M. hyopneumoniae*, *M. flocculare* and *M. hyorhinis* has been recently described [6], showing highly levels of similarity in the genome contents and organizations of *M. hyopneumoniae* and *M. flocculare*. These two closely related mycoplasma species [5], [6] present with pathogenic differences; *M. hyopneumoniae* is considered to be pathogenic, and *M. flocculare* is considered to be a commensal species. This genome comparison showed many differences that may help to explain their differing behaviors. However, they were not able to identify specific virulence determinants that could explain the differences in their pathogenicities [6]. This same comparative analysis showed that the *M. hyorhinis* genome also shares significant numbers of genes with both *M. hyopneumoniae* and *M. flocculare*. The differences in *M. hyorhinis* gene composition seem to be related mainly to the ability of *M. hyorhinis* to colonize different hosts and sites.

### Transcriptional Units structures

**Figure 2:**
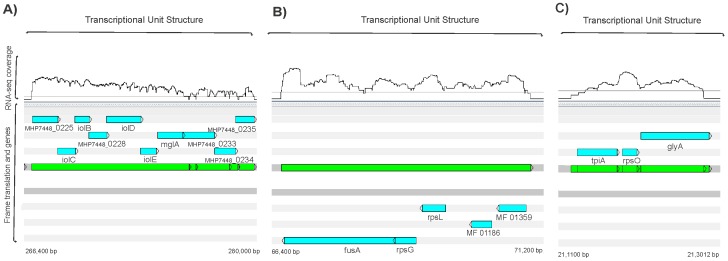
Transcriptome feature in the reference condition. The light blue arrows represents the predicted genes and they are positioned at the direction of transcription. The gene names are presented below the arrows. The green arrows represents the mRNA transcript. (**A**) *M. hyopneumoniae* Myo-inositol transcription unit (TU) composed by ten genes. This TU is on the forward strand in the genome and has a staircase behavior, meaning that the consecutive genes have lower and steady expression levels. (**B**) *M. flocculare* TU structure composed by five genes. This TU is located on the reverse strand in the genome. (**C**) *M. hyorhinis* TU structure composed by three genes. This TU is located on the forward strand in the genome. The position on the chromosomal sequence is indicated in base pairs (bp) below both termini of the bars. Visualization by the software Artemis.

We have constructed transcriptome maps of *M. hyopneumoniae*, *M. flocculare* and *M. hyorhinis*. The RNA-Seq data enabled us to determine the structures of the TUs on a genome-wide scale, which is important for the identification of co-expressed genes and for the understanding of the coordinated regulation of the mycoplasma transcriptome. Our results support previous predictions that the genes are preferably transcribed in polycistronic mRNA, forming TUs, in the mycoplasma genomes [6], [18]. We have identified that co-expression occurs for approximately 90% of the predicted genes for all three species (Table 1; Table S5; Table S6; Table S7). By joining consecutive overlapping reads or reads that mapped uniquely (Figure 2), we have constructed a common scheme of gene transcription in mycoplasmas. Recently, a comparative genome analysis of *M. hyopneumoniae*, *M. flocculare* and *M. hyorhinis* was described [6]. The arrangements of the TUs as determined by *in silico* prediction revealed that they are present in similar numbers in these three mycoplasmas genomes [6], indicating that genes are preferably organized into TUs in *M. flocculare* and *M. hyorhinis* similar to *M. hyopneumoniae* [18]. Moreover, as previously described for *M. hyopneumoniae*, the overall gene distributions within the TUs in *M. flocculare* and *M. hyorhinis* are highly variable with respect to gene number and the functional categories of the encoded products [6]. As expected, these TUs predicted by *in silico* and *in vitro* approaches in *M. hyopneumoniae* in addition to the computationally predicted TUs in the *M. flocculare* and *M. hyorhinis* genomes were confirmed in the three transcriptomes analyzed here.

Over 80% of the TUs predicted for the three species were either confirmed or partially confirmed by our transcriptome analysis (Table 1; Table S5; Table S6; Table S7). Table 1 shows the confirmed TUs, for which all ORFs exhibited cotranscription, besides showing the partially confirmed TUs, for which the majority of the ORFs exhibited cotranscription. Among the predicted TUs for *M. hyopneumoniae* and *M. hyorhinis* [6], [18], only two were not associated with any transcriptional products in the present transcriptome maps. All genes belonging to these TUs, in both genomes, encoded hypothetical products. Remarkably, in the *M. flocculare* transcriptome, all of the TUs predicted by the previous *in silico* analysis [6] possessed at least one gene that was transcribed (Table 1).

The overlapping reads mapping to each reference genome generated a surprisingly large number of long contigs (Figure S2). Some examples are the contigs that mapped to nucleotides 268881–277986 with sizes of over 9,000 bp, and the mapped contig at position 242981–245879 with a total length of 2,898 bp, which were both observed in *M. hyopneumoniae* 7448. The *M. hyorhinis* map has the largest mRNA contig mapped, with a size reaching of 13,000 bp (locations: 485595–498603 bp and 779997–788652 bp). Figure 2 illustrates a representative TU for each of the analyzed transcriptomes. Most of the observed TUs possessed overlapping reads (as shown in Figures 2a, 2b and 2c, green arrows). Interestingly, the transcript levels of genes belonging to the same TU were variable (see Figures. 2a, 2b and 2c), indicating that such staircase-like expression is a widespread phenomenon in bacteria. The presence of consecutive genes within operons possessing different expression levels has been observed in other bacterial species [21], [65]. The *M. pneumoniae* transcriptome data showed a natural polarity with respect to the TU, in which the first gene of the TU exhibits the most prominent level of transcription, and levels progressively decreased for each successive gene in the TU [21]. Thus, although genome reduction leads to longer operons accommodating genes with different functions [18], [21], [66], the last gene can still retain internal initiation and termination sites under certain conditions.

Mycoplasmas are characterized by the presence of long TUs containing genes that encode highly functionally variable products. Moreover, all the *M. hyopneumoniae* TUs are preceded by putative promoter sequences [20], providing evidence of gene organization as an important factor in the regulation of transcription. A subset of genes within TUs that are able to be transcribed by alternative internal promoters have been demonstrated and associated with possible complex transcriptional organization in *M. hyopneumoniae* genomes [18]–[20]. According to previous predictions [20], 70% of *M. hyopneumoniae* TUs possess internal promoter sites, which is in agreement with the high variations in expression levels that were observed within the TUs. These findings are also similar to others that have been described in many bacterial species, including those in the *M. pneumoniae* transcriptome, which has been characterized by the frequent occurrence of polycistronic operons with alternative transcripts [21], and the *Bacillus subtilis* transcriptome, in which 20% of the genes in polycistronic operons are transcribed from more than one promoter [67]; additionally, approximately 6% of the polycistronic operons contain internal read-through terminators, at which partial continuation of transcription occurs [68]. Similarly, in the *Halobacterium salinarum*, 40% of the operons are expressed in condition-dependent manners [69]. These findings indicate that various activators and/or repressors can regulate transcription. Thus, a TU could respond to different inputs, enabling higher regulatory responsiveness [33]. Taken together, these data suggest a complex relationship between genomic organization and gene expression in prokaryotes.

Overall, we provided comprehensive assessments of the transcriptomes of three important *Mycoplasma* species that inhabit the respiratory tract of swine. We have analyzed the RNA populations present in *M. hyopneumoniae*, *M. flocculare* and *M. hyorhinis* in detail and used the data to map transcript boundaries and operon structures on a genome-wide scale. Moreover, we showed that almost all of the genes in these mycoplasma genomes are expressed at some basal level and that the majority of the genes are co-expressed, confirming the previously predicted TUs. Our results expand upon current knowledge regarding the coordination of transcription in swine respiratory mycoplasmas, in which genes are preferably transcribed in TUs. The determinations of all of the functional elements in the *M. hyopneumoniae*, *M. flocculare* and *M. hyorhinis* is a prerequisite for formulating holistic approaches to delineating the complex transcriptional regulation that occurs in these mycoplasma species.

## Supporting Information

Figure S1 **Genome wide assessment.** Circular plot of the reads mapping to the (**A**) *M. hyopneumoniae* 7448, (**B**) *M. flocculare* ATCC 27716 and (**C**) *M. hyorhinis* HUB-1 genomes. The outer circle (black) is marked in basepairs. The outermost circles represent CDS on the forward (outermost) and reverse (second outermost) strand (blue). Coding regions are illustrated by intermediate gray circle. The green inner circle represents the mapped sequence reads with a minimum quality score of 20. Visualization by DNAplotter.
(TIF)

Figure S2 **Length distribution of contigs from** ***M. hyopneumoniae*** **(MHP),** ***M. flocculare*** **(MFL) and** ***M. hyorhinis*** **(MHR) transcriptome assembly.**
(JPG)

Table S1 **Transcriptome mapping from** ***Mycoplasma hyopneumoniae*** **.**
(XLSX)

Table S2 **Transcriptome mapping from** ***Mycoplasma flocculare*** **.**
(XLSX)

Table S3 **Transcriptome mapping from** ***Mycoplasma hyrhinis*** **.**
(XLSX)

Table S4 **Probably noncoding RNA detected in the three transcriptome studied.**
(XLSX)

Table S5 ***Mycoplasma hyopneumoniae*** **7448 trancriptional units and cotranscription analysis.**
(XLS)

Table S6 ***Mycoplasma flocculare*** **trancriptional units and cotranscription analysis.**
(XLS)

Table S7 ***Mycoplasma hyorhinis*** **trancriptional units and cotranscription analysis.**
(XLS)
