# Correction: Unravelling the Transcriptome Profile of the Swine Respiratory Tract Mycoplasmas

**DOI:** 10.1371/journal.pone.0116122

**Published:** 2014-12-19

**Authors:** The PLOS ONE Staff

Table S2 is a duplicate of Table 2. Please view the correct Table S2 below.

## Supporting Information

Table S2 Transcriptome mapping from *Mycoplasma flocculare.*
(XLSX)
