# Supplementary figures and images for: Unravelling the Transcriptome Profile of the Swine Respiratory Tract Mycoplasmas
Source: PLoS One. 2014 Oct 15;9(10):e110327. doi: 10.1371/journal.pone.0110327 (PMC4198240; doi:10.1371/journal.pone.0110327)

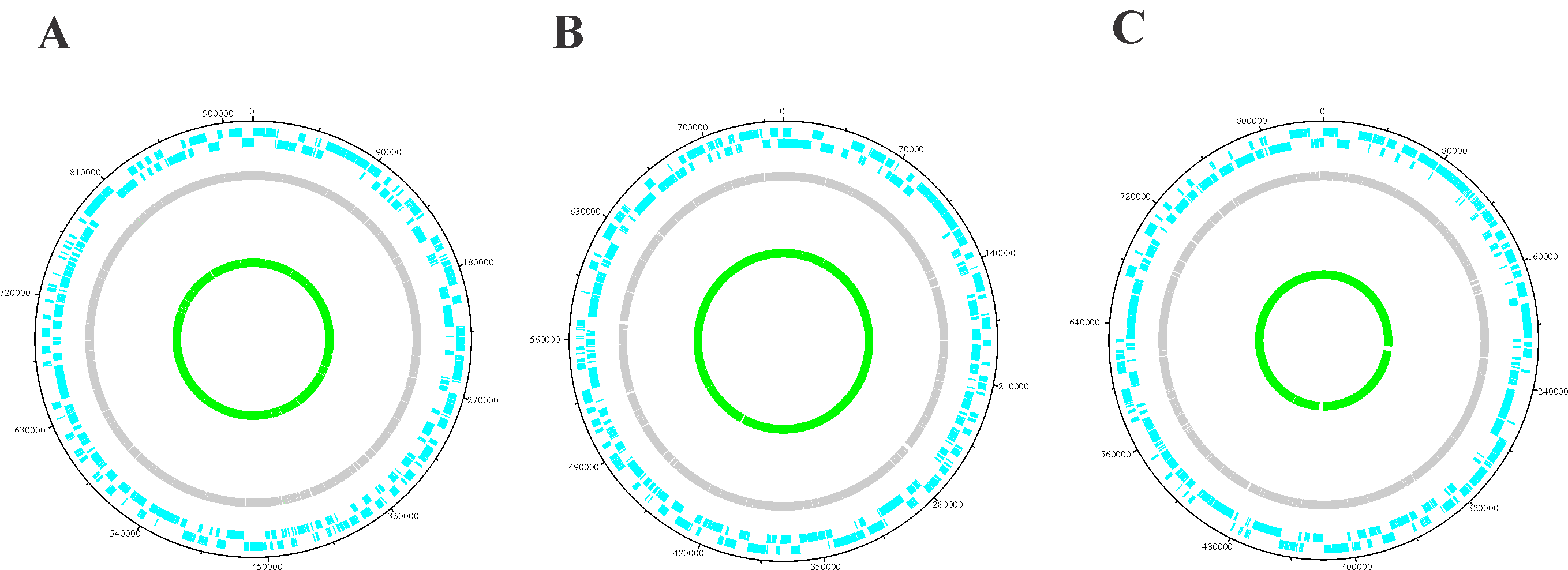

Supplement: Figure S1 — Genome wide assessment. Circular plot of the reads mapping to the (A) M. hyopneumoniae 7448, (B) M. flocculare ATCC 27716 and (C) M. hyorhinis HUB-1 genomes. The outer circle (black) is marked in basepairs. The outermost circles represent CDS on the forward (outermost) and reverse (second outermost) strand (blue). Coding regions are illustrated by intermediate gray circle. The green inner circle represents the mapped sequence reads with a minimum quality score of 20. Visualization by DNAplotter. (TIF) [file pone.0110327.s001.tif]

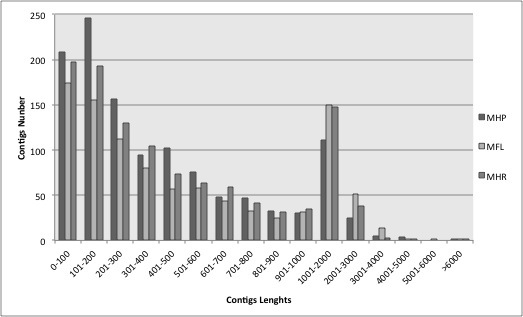

Supplement: Figure S2 — Length distribution of contigs from M. hyopneumoniae (MHP), M. flocculare (MFL) and M. hyorhinis (MHR) transcriptome assembly. (JPG) [file pone.0110327.s002.jpg]
